# Li Fraumeni Syndrome predisposes to gastro-esophageal junction tumours

**DOI:** 10.1007/s10689-023-00353-0

**Published:** 2024-01-11

**Authors:** Douglas Tjandra, Alex Boussioutas

**Affiliations:** 1 Familial Cancer Centre, The Peter MacCallum Cancer Centre, Melbourne, VIC Australia; 2 Department of Gastroenterology, Alfred Hospital, 99 Commercial Rd, Melbourne, VIC 3004 Australia; 3 Department of Gastroenterology, Central Clinical School, Monash University, Melbourne, VIC Australia

**Keywords:** Li Fraumeni syndrome, *TP53*, hereditary gastric cancer syndromes, Endoscopic surveillance

## Abstract

Li-Fraumeni Syndrome (LFS), caused by germline pathogenic variants in *TP53*, predisposes to a wide range of young-onset malignancies, particularly sarcoma, breast and brain cancer. More recently, an increased risk of gastric adenocarcinoma has been recognised, although uptake of surveillance upper endoscopy is unclear. Our retrospective review of 65 patients with LFS, of whom 53.8% had undergone endoscopy, identified four patients (6.2%) with gastro-esophageal junction (GEJ) adenocarcinomas. Two cases were found on asymptomatic screening and were early stage. No cases had family history of gastrointestinal malignancy. Reviewing genomic data from The Cancer Genome Atlas Program, 76.4% of sporadic esophageal adenocarcinomas harboured somatic *TP53* pathogenic variants, compared with 39.9% of non-cardia gastric cancers. This similar pattern observed in germline and sporadic cases warrants further investigation. We propose that upper endoscopy be recommended to all patients with LFS, with a focus on appropriate surveillance of the GEJ.

**Supplementary Information:**

The online version contains supplementary material available at 10.1007/s10689-023-00353-0.

## Introduction

Li-Fraumeni Syndrome (LFS) is defined by germline pathogenic variants in the tumour suppressor gene *TP53* and is associated with increased rates of many different malignancies, some with up to 100% lifetime risk without appropriate intervention [1]. While the strongest associations have been demonstrated with sarcoma, breast cancer, brain tumours and adrenocortical carcinomas, the increased risk of colorectal and, more recently, gastric adenocarcinomas have also been acknowledged [2]. The relative risk of esophageal cancer is less well characterised with conflicting reports [1, 3]. An analysis of the International Agency for Research on Cancer database identified 0.5% of individuals with esophageal cancer (15/3043) and 3.3% (101/3043) with gastric cancer [3, 4]. Gastro-esophageal junction (GEJ) tumours arise at the histological transition between esophagus and stomach, and the commonly used Siewert classification divides GEJ adenocarcinomas into three categories for which oncological management differs: Type 1 arising from distal esophagus (1 to 5 cm proximal to GEJ), Type 2 located at the true junction (between 1 cm proximal to 2 cm distal of the GEJ) and Type 3 located 2 to 5 cm distal to the GEJ [5]. In contrast, the American Joint Committee on Cancer stages all GEJ tumors with epicentre ≤ 2 cm into the proximal stomach as esophageal cancers and those > 2 cm as gastric cancers [5]. Consequently, GEJ tumors have been variably categorised in the literature as esophageal, gastric or a separate entity.

In this context, expert consensus guidelines differ in their recommendations for upper endoscopy, or esophagogastroduodenoscopy, screening in LFS: North American guidelines support upper endoscopy every 2–5 years from the age of 25 alongside colonoscopy, while European guidelines do not routinely recommend upper endoscopy screening [2, 6]. The uptake of these recommendation is also unclear.

There is a biological basis to suspect pathogenic variants in *TP53* as a driver of upper gastrointestinal cancers. The Cancer Genome Atlas Program classified 559 sporadic esophageal and gastric cancers into distinct molecular phenotypes. When analysing adenocarcinoma, four subclasses were identified, but only one, a chromosomal instability (CIN) subclass, accounted for the vast majority of lower esophagus and GEJ tumours [7]. CIN molecular subgroup accounted for 49% of adenocarcinomas from esophagus and stomach and was characterised by *ERBB2* amplification, *VEGFA* amplification and, importantly, pathogenic variants in *TP53*.

The familial cancer centre at our institution offers a dedicated gastrointestinal risk management clinic for patients with high-risk genetic predisposition to gastrointestinal malignancy, and we routinely offer upper endoscopy at the same time as colonoscopy to patients with LFS. We sought to assess the rates of upper gastrointestinal cancers and their characteristics in our cohort, with comparison to results from the TCGA cohort.

## Methods

A retrospective chart review was performed of adult patients with clinical class 4 or 5 germline pathogenic variants in *TP53* managed by our centre between January 2000 to May 2023. Data regarding demographics, personal and family history of malignancy, duration of follow-up, and endoscopic/histologic results of upper and lower endoscopies were collected. Further clinical details were collated for cases of GEJ tumours. Ethical approval was obtained from the institutional human ethics committee (Project number: PMC97246, 29 May 2023).

Separately, data of each subject analysed in the TCGA study to characterise the molecular characteristics of sporadic esophageal and gastric cancers was obtained from the supplementary table in the initial publication. Corresponding details of pathogenic variants in *TP53* were then obtained from the openly available dataset on cBioportal (accessed 27 June 2023) [7, 8]. From 559 subjects, 90 esophageal squamous cell cancers, 13 gastric adenocarcinomas of unclear location and 2 undifferentiated esophageal tumours were excluded. The remaining 454 cases of adenocarcinoma were then subdivided based on subtype (esophageal/probable esophageal GEJ adenocarcinoma, indeterminate GEJ adenocarcinoma, gastric/probable gastric GEJ adenocarcinoma) and proportion of cases with somatic pathogenic variants in *TP53* calculated.

## Results

**Fig. 1:**
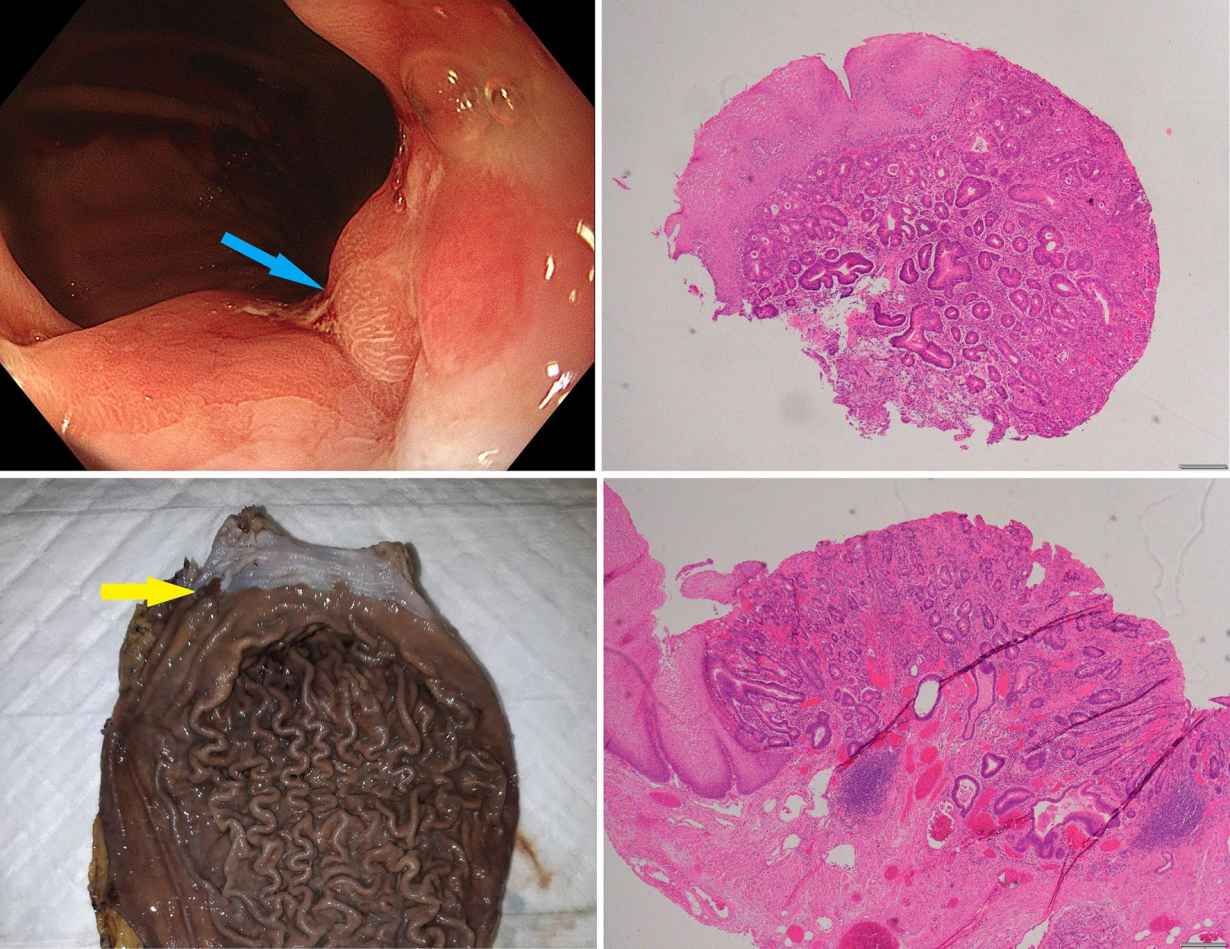
Panel of endoscopic, surgical and histologic findings in one case of GEJ adenocarcinoma on screening: Top, left: a subtle GEJ nodule at endoscopy (blue arrow); Top, right: endoscopic biopsy (H&E stain, x40) demonstrating adenocarcinoma adjacent to squamous mucosa; Bottom, left: surgical resection specimen with irregularity at GEJ (yellow arrow); Bottom, right: resection histology (H&E stain, x40) demonstrating adenocarcinoma confined to the mucosa, at the GEJ

Sixty-five with LFS (57% female, mean at diagnosis 34.5, median follow-up 51 months) seen via the high-risk cancer genetics clinic over the study period, 35 (53.8%) of whom had at least one upper endoscopy. Four patients (6.2%) were diagnosed with cancer at the GEJ (Table 1), with no cancers elsewhere in the upper digestive tract and one separate case of colorectal cancer on colonoscopy. Two cases, a White man and North-East Asian woman in their 30s, were asymptomatic and undergoing screening, at their second and first procedures respectively. Both cancers were at early stage allowing for resection with curative intent. The lesions were subtle and best appreciated on close inspection of the GEJ in forward-view (Fig. 1). In the former case, index upper endoscopy revealed moderate reflux esophagitis (Los Angeles Classification grade B) with no dysplasia on biopsy, but a decision was made for early repeat surveillance at 18 months revealing subsequent carcinoma in situ. No cases of cancer were associated with prior family history of digestive tract malignancy, or abdominal radiotherapy. Furthermore, five patients undergoing screening (14.3% of those who had upper endoscopy) were diagnosed with a premalignant lesion (Barrett’s esophagus, non-*Helicobacter* associated gastric intestinal metaplasia and a proximal fundic gland polyp with low grade dysplasia).

**Fig. 2:**
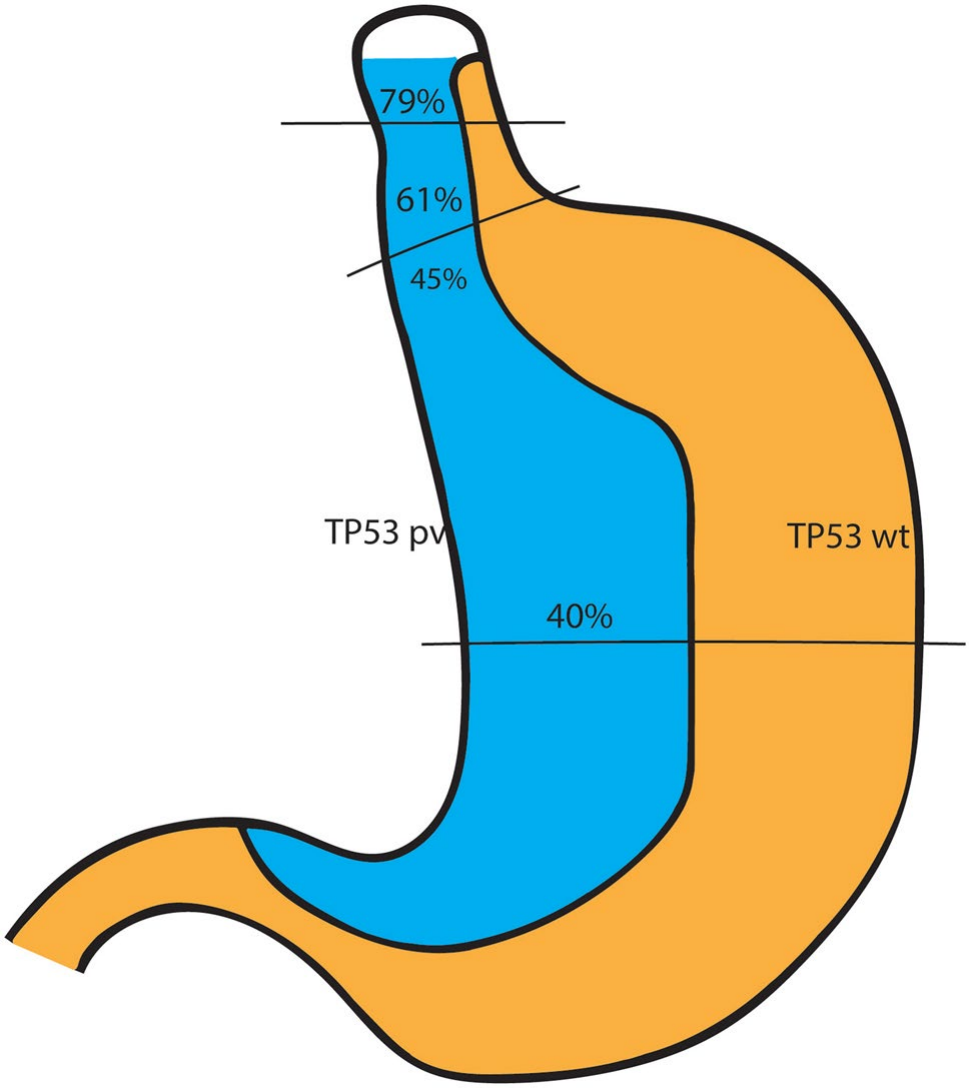
Relative proportions of somatic pathogenic variants (pv) and wild type (wt) in TP53 in each adenocarcinoma subgroup in the TCGA cohort. Esophageal/probable esophageal, indeterminate and gastric/probable gastric groups were GEJ tumours

On review of the data for these patients from the TCGA paper via cBioportal, we found that amongst esophageal or probable esophageal adenocarcinomas, 76.4% (55/72) had somatic pathogenic variants in *TP53*, with decreasing prevalence in the indeterminate GEJ (22/36, 61.1%), gastric/probable gastric GEJ (29/63, 46.0%) and gastric non-cardia (113/283, 39.9%) adenocarcinoma groups (Fig. 2).

## Discussion

It is still not globally recognised that upper endoscopy should be a routine component of surveillance in LFS. Moreover, in most guidelines recommending upper endoscopy, the focus has been on gastric adenocarcinoma, perhaps because some reports have presumably grouped GEJ cancers with gastric cancers [9], even though some cases are better characterised as esophageal or a distinct entity [5, 7]. The distinction is important as inspection of the gastric mucosa is performed within the stomach and on retroflexion during upper endoscopy, while the GEJ is best assessed in forward-view.

**Table 1:** Characteristics of malignant and premalignant lesions on upper endoscopy of LFS cohort

**Age (years)**	**Sex**	**Ethnicity**	**Genetic testing for LFS**	Nucleotide variant in *TP53*	**Personal history– malignancy (age, years)**	**Family history–GI malignancy (age, years)**	**Indication for EGD**	**Number of prior EGD** **(months from previous)**	**Endoscopic finding**	**Histologic finding**	**Outcome**
46	Male	White	Predictive	c.586C>T (exon 6)	Nil	Nil	Reflux	0 (n/a)	GOJ mass	Adenocarcinoma	Metastatic disease
52	Male	White	Proband	c.743G>T (exon 7)	Sarcoma (45)	Nil	Dysphagia	0 (n/a)	GOJ mass from distal esophagus to fundus	Adenocarcinoma	Metastatic disease
28	Male	White	Predictive	c.659A>G (exon 6)	Nil	Nil	Surveillance	1 (18)	Esophagitis Barrett’s 2x 3mm mucosal nodules at GOJ	Carcinoma in situ with submucosal extension	Endoscopic mucosal resection without recurrence
37	Female	North-East Asian	Proband	c.742C>T (exon 7)	Breast (35)	Nil	Screening	0 (n/a)	Esophagitis Subtle erosion at GOJ	Adenocarcinoma	No distant metastases, underwent extended total gastrectomy
34	Male	White	Predictive	c.742C>T (exon 7)	Nil	Nil	Screening	0 (n/a)	Polyp in fundus	Eosinophilic esophagitis. Fundic gland polyp with low grade dysplasia.	Resected. Ongoing surveillance.
33	Male	White	Predictive	c.473G>A (exon 5)	Nil	Gastric (40s), colorectal (40s)	Reflux	1 (27)	Antral gastritis	Antral-limited IM	Ongoing surveillance.
36	Female	White	Proband	c.473G>A (exon 5)	Nil	Gastric (age unclear), colorectal (40)	Screening	0 (n/a)	Gastric fundic gland polyps. Antral gastritis.	Antral IM	Ongoing surveillance.
32	Male	South Asian	Proband	c.841G>A (exon 8)	Sarcoma (32)	Nil	Reflux	0 (n/a)	Antral gastritis. Barrett’s esophagus.	Antral and esophageal IM	External follow-up.
18	Male	White	Predictive	c.794T>C (exon 8)	Nil	Colorectal (20)	Screening	0 (n/a)	Esophagitis. 1mm nodule at GOJ.	Foveolar hyperplasia and IM (GOJ). Antral-limited IM.	Treated with proton-pump inhibitor. Regression of changes on follow-up endoscopy.

*EGD*  Esophagogastroduodenoscopy

Our findings suggest that the GEJ and lower esophagus may be a particularly vulnerable region in LFS, in keeping with the molecular phenotyping seen in sporadic GEJ tumours. While it has been suggested that Asian carriers may be at higher risk than non-Asian carriers, and that risk of both gastric and colon cancers may run in families [9, 10], the risk in our cohort was not isolated to these specific groups. Both cases seen on surveillance were early stage, facilitating likely curative resection. This data suggests a harmonisation of germline and somatic observations that are highly suggestive of a biological effect of p53 involving the GEJ. There are limitations to the data given this is a retrospective analysis of a cohort of LFS, but the high prevalence of GEJ cancer in this group is compelling and warrants a prospective evaluation in patients who carry germline pathogenic variants of *TP53*.

Additionally, premalignant lesions in the upper gastrointestinal tract appeared relatively frequently in our cohort. Given the presence of gastric intestinal metaplasia in four of the five individuals with premalignant lesions, we would also endorse routine biopsy of the gastric antrum and body and eradication of *Helicobacter pylori* if present, similar to recommendations for Lynch Syndrome [11].

The optimal interval for upper endoscopy also requires further study. The progression from reflux esophagitis to carcinoma in situ occurred in 18 months in one case, and a longer surveillance interval may have led to a later stage diagnosis. Notably, this finding would not have been routinely followed-up in a patient with no germline pathogenic variant in *TP53*. It may therefore be reasonable for any endoscopic finding at index procedure to be followed-up at two years, and subsequent follow-up guided by the progression or resolution of the changes. Our practice, in the absence of data, is for patients with other risk factors such as family history of gastrointestinal malignancy or abdominal radiotherapy to undergo two to three yearly surveillance, generally coinciding with colonoscopy. Patients with normal index upper endoscopy and no other risk factors undergo surveillance every five years.

We believe our novel clinical observations provide practice-changing evidence for all clinicians caring for patients with germline pathogenic variants of *TP53* and we would advocate for routine endoscopic surveillance in all cases, with particular focus on the GEJ.

### Supplementary Information

Below is the link to the electronic supplementary material.

Supplementary material 1 (DOCX 33003.2 kb)

## Data Availability

The retrospective clinical dataset generated during and/or analysed during the current study is not publicly available due privacy requirements but relevant data is available from the corresponding author on reasonable request.
